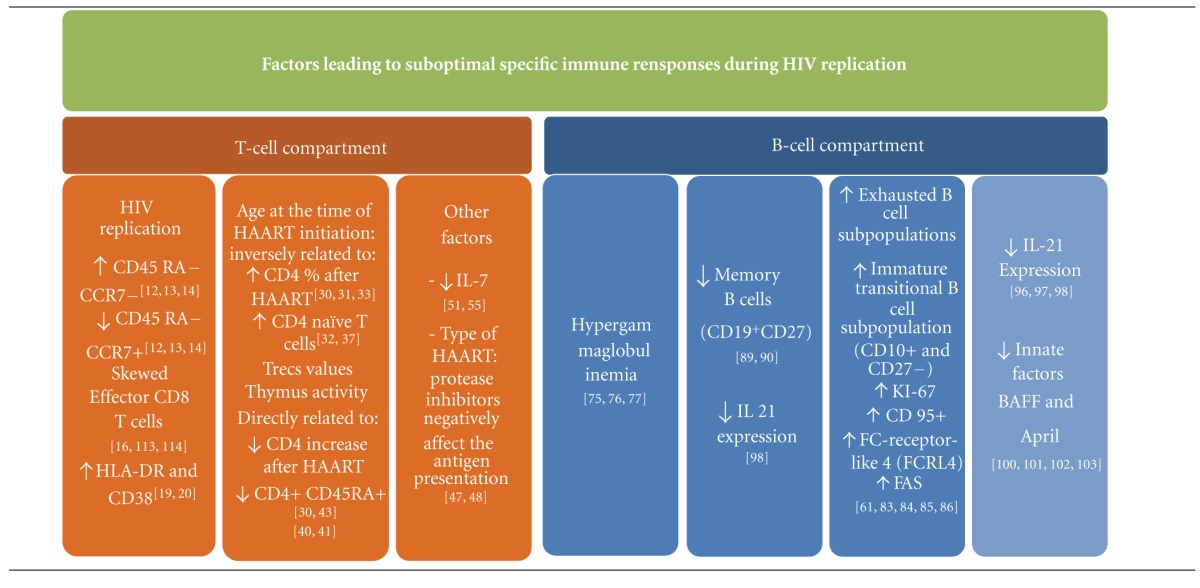# Suboptimal Immune Reconstitution in Vertically HIV Infected Children: A View on How HIV Replication and Timing of HAART Initiation Can Impact on T and B-cell Compartment

**DOI:** 10.1155/2012/805151

**Published:** 2012-04-08

**Authors:** Nicola Cotugno, Iyadh Douagi, Paolo Rossi, Paolo Palma

**Affiliations:** ^1^University of Rome, Tor Vergata, 00133 Rome, Italy; ^2^Department of Medicine, Karolinska Institute, SE-141 86 Stockholm, Sweden; ^3^University Department of Pediatrics, DPUO, Children' Hospital Bambino Gesù, Piazza S.Onofrio, 4-00165 Rome, Italy

## Abstract

Today, HIV-infected children who have access to treatment face a chronic rather than a progressive and fatal disease. As a result, new challenges are emerging in the field. Recent lines of evidence outline several factors that can differently affect the ability of the immune system to fully reconstitute and to mount specific immune responses in children receiving HAART. In this paper, we review the underlying mechanisms of immune reconstitution after HAART initiation among vertically HIV-infected children analyzing the possible causes of suboptimal responses.

## 1. Introduction

Highly active antiretroviral treatment (HAART) has dramatically changed the course of HIV infection, allowing control of viral replication and the restoration of immune function [1]. However, the success currently experienced in many patients receiving HAART remains far from universal or permanent. Children who have been highly compliant to HAART at younger ages frequently present adherence problems during adolescence [2]. Recent data clearly show how, after five years of continuous HAART, vertically HIV-infected children are at a high risk of developing triple-class virological failure [3]. New lines of evidence outline several factors that can differently affect the ability of the immune system to fully reconstitute and maintain specific immune responses in children under HAART. A better understanding of how HAART affects immunity is needed. Here, we review present knowledge regarding immunity in HIV-infected children, exploring the impact of HIV viral load, HAART, timing of initiation, and age on B- and T-cell recovery and maintenance. In addition, we describe immune responses to vaccinations as a model system to review possible causes of immune memory dysfunction and suboptimal reconstitution in vertically HIV-infected children on HAART.

## 2. T-Cell Compartment and HAART

With initiation of HAART, immune activation declines in parallel to the reconstitution of naïve and memory T-cell subsets [3–6]. Apparently, three mechanisms play a key role in T cell immune reconstitution process in HIV-infected individuals. De novo production by the thymus plays a crucial role in the rise of mostly naïve CD4^+^ in younger patients [6–9], whereas an increase in CD4^+^ T cell half-life and homeostatic proliferation by the residual memory CD4^+^ T cells are predominant mechanisms in older subjects [10]. The ability of the immune system to develop and maintain specific immune responses will depend on the predominance of one of these mechanisms. In fact, even if an absolute CD4^+^ T-cell count can be fully restored, T-cell immune reconstitution can be “partial” if it is based on the production of new CD4^+^ T-cell or “truncated” if it is mainly from the remaining repertoire of CD4^+^ T cell [11]. Factors such as age, viremia, timing of HAART initiation and involution of the thymus can play a critical role in this process leading to quantitative and qualitative differences in the immune reconstitution.

## 3. Factors Leading to Suboptimal Reconstitution of T-Cell Compartment in HIV-Infected Children on HAART

### 3.1. HIV Viremia

HIV causes qualitative and quantitative dysfunctions of T-cell compartment in both CD4^+^ and CD8^+^ subsets. Under viral replication, naïve CD4^+^ and CD8^+^ T-cells are stimulated to enter the circulation and differentiate into effector memory (CD45RA^+^ CCR7^−^) and effector phenotype (CD45RA^−^ CCR7^−^), while central memory (CD45RA^−^ CCR7^+^) compartment is depleted [12–14]. However, persistent exposure to high levels of viremia results in a dysfunctional immune-specific response to HIV leading to exhaustion of naïve CD8 T-cells and skewed maturation of memory subsets [15, 16]. Virus-specific CD8 T-cell exhaustion is characterized by the incremental loss of proliferative and effectors properties [17, 18]. Moreover, a continued antigenic stimulation induces an increased expression of surface activation markers, such as HLA-DR and CD38 [19, 20]. A positive relation between the expression of these markers and CD4^+^ and CD8^+^ depletion has been reported [21] and directly related to clinical disease progression in both HIV-infected adults and infants [22, 23]. Persistent HIV viremia has also been related to an increase in T cell apoptosis. A higher expression of the key regulatory marker of apoptosis (CD95) on CD4^+^ has been described during HIV infection [24–26]. Conversely, a significant decrease in CD95 expression, with the reduction of CD4^+^ and CD8^+^ T cells apoptosis, has been observed after HAART initiation in HIV-infected children and adolescents [27]. However, since the reduced apoptosis is restricted to the CD45RO-positive (primed/memory) T-cells subpopulation, the simultaneous increase in circulating resting/naïve T cells observed in pediatric patients can be explained by the new generation of naïve T cells from the thymus.

### 3.2. Quality of Reconstitution: Age Makes the Difference

Previous studies among transplant and chemotherapy recipients indicated that age directly influences immune reconstitution [28, 29]. In these patients, CD4^+^ T naïve or memory expansion specifically contributing to the immune reconstitution ultimately differs according to age.

Similarly, a direct relation between the individuals age, naïve T-cell emigration, and memory T-cell expansion has also been demonstrated in vertically HIV-infected children after HAART initiation [30–33]. Since the patient's age can have an impact on immune reconstitution after HAART initiation, the age of HIV transmission and timing of HAART initiation must be carefully considered [34]. In a cohort of 265 HIV-infected children naïve to treatment, Walker et al. found that the short-term (6 months) CD4% increase after HAART initiation was positively related to younger age and inversely related to pre-HAART CD4% [35]. In addition, several authors reported that immune restoration in infants mainly involves naïve cells, while it mostly relies on expansion of memory T cells in older children [30, 33, 35]. Indeed immune recovery that follows HAART initiation is faster in younger children compared to older ones or adults [36–39]. As shown by multiple studies analyzing thymic output using T-cell receptor excision circle (TRECs) assays [40], thymic function plays a pivotal role in this process [41]. Physiologically, thymus function is inversely related to age. It has been shown that HIV-1-infected children present lower TREC values than health-matched controls and a significant increase in parallel with CD4^+^ count after HAART initiation, particularly at younger ages [42]. Thus, to warrant an optimal immune reconstitution, WHO 2010 recommendations suggest the initiation of HAART in all HIV-infected children between two and five years with either a CD4^+^ count of 750 cell/mm^3^ or below, or a CD4^+^ percentage of 25 or below, whichever is lower, irrespective of clinical status [43].

The relation between age and control of viremia has also been extensively addressed by several authors [30, 34, 39, 44]. Untreated newborns and infants present an higher peak of viremia during acute infection. In addition, younger patient's age has been related to the slower achievement of viral control compared to older one despite effective HAART [45, 46]. The immaturity of the immune system, together with differences in pharmacokinetics and pharmacodynamics of antiviral drugs, may account for a less efficient containment of HIV viral replication during infancy [30].

### 3.3. Other Possible Factors Influencing Suboptimal T-Cell Responses

#### 3.3.1. Use of Different Antiretroviral Drugs Classes

First-line boosted protease inhibitors (PI) regimens have been recently shown to be equally effective than Nonnucleoside reverse transcriptase inhibitors (NNRTI) ones in terms of immune reconstitution and long-term control of viremia in HIV-infected children [36]. However, small observational studies in vertically HIV-infected children show increased HIV-specific cellular immune responses after switching from a PI to an NNRTI-based regimen [47, 48]. A possible relation between the use of different antiretroviral drug classes and the ability of the immune system to mount T-cell-specific immune responses has been proposed. Several authors have suggested that PI may cause immune suppression by interfering with antigen presentation. In vitro studies [49] showed the ability of PI drugs to modulate proteasome peptidase activity and cause intracellular accumulation of ubiquitin tagged proteins. Increased HIV-specific immune T-cell response, in terms of lymphoproliferation and intracellular IFN-*γ* and tumor necrosis factor-*α* production, has been described in HIV-infected children who changed to a PI-sparing therapy owing to failure of viral control or due to toxicity [47, 48].

#### 3.3.2. Levels of IL-7

The IL-7/IL-7R pathway has been shown to play a key role in sustaining peripheral CD4^+^ T-cell homeostasis [50, 51].

High levels of IL-7 were demonstrated among HIV-infected adults in association with higher CD4^+^ depletion [52, 53]. However, data on IL-7 activity in HIV-infected children are discordant and still unclear. The presence of a better higher thymus activity at younger ages may lead to a faster T-cell turnover, resulting in different IL-7 consumption [54, 55]. Therefore, lower IL-7 levels observed among HIV-infected children may result from an increased consumption of IL-7 by newly produced T cells in response to active viral replication. In line with this observation, a strong relation between low levels of IL-7 and extent of HIV viral replication has also been established [56]. Taken together, this may suggest that low levels of IL-7 could be a predictable marker of virological failure in HIV-infected children [55, 56].

## 4. B-Cells Compartment and HAART

A decline in total CD27^+^ memory B-cells, hypergammaglobulinemia [57–59], impaired reactivity or loss of specific antibodies gained during the normal vaccination schedule, increased expression of markers activation [60–62], high spontaneous autoantibody production in vitro [63], and an increased incidence of B-cell malignancies [64] have all been reported as direct and indirect consequences of HIV infection [65] (Table 1).

Hypergammaglobulinemia reflects a generalized HIV-1-driven polyclonal B-cell activation and was shown to be directly related to viremia and inversely related to CD4% [66]. After virologic suppression with HAART, CD4^+^ T-cell count increases, hypergammaglobulinemia decreases to normal levels, and an increase in the absolute CD19^+^ B-cell count has been observed [67–71].

Furthermore, HAART permits the reduction of B-cells subpopulations that are abnormally expanded during ongoing HIV replication and are prone to apoptosis [72]. In a previous study, we observed a decrease of immature transitional B-cells after achievement of viral suppression through HAART in vertically infected children [54]. Similarly, an expanded population of immature transitional B-cells during uncontrolled viremia and their normalization after HAART initiation has been described in adults [61, 73].

These results suggest that effective HAART permits the normalization of B-cell subpopulations and apoptosis-prone B-cell reduction; however, the number and magnitude of B-cell alterations and their qualitative function caused by HIV replication cannot be fully restored by HAART [74, 75]. 

The following is a review of the factors leading to a suboptimal response of B-cell compartment, focusing on the impaired ability of the immune system to develop an effective B-cell response to infectious agents or vaccinations in HIV-infected children on HAART.

## 5. Factors Leading to Suboptimal Response of B-Cell Compartment in HIV-Infected Children on HAART

### 5.1. The Impact of Active HIV Replication

#### 5.1.1. Hypergammaglobulinemia

Aberrant activation of the B-cell compartment and hypergammaglobulinemia were among the first recognized characteristic of HIV-1-infected individuals [76, 77]. Although not yet fully matured in children, B-cells are already subject to HIV-induced immune activation. Hypergammaglobulinemia and reduced antigen-specific humoral responses in HIV-infected children were first described by Bernstein LJ in 1985 [78]. However, mechanisms responsible for hyperimmunoglobulinemia in HIV infection are unclear. An increased frequency of B-cells secreting high levels of Ig during viremia has been attributed to the expansion of the CD21^low^ B-cell subpopulation in the peripheral blood of HIV-viremic patients. In addition, it has been recently shown that the ligation of virion-associated host CD40L with the cell surface CD40 is sufficient to efficiently activate B-cells in a polyclonal fashion [79]. Whereas it has been reported that viral proteins, such as Nef [80, 81] and gp120 [82, 83], can act as triggers for Ig production and dysfunctional switch through a CD40-independent pathway.

#### 5.1.2. Abnormal Expansion of B Cell Subpopulations

During HIV infection, different B-cell subpopulations are irreversibly damaged, and markers present on their surface make them dysfunctional and prone to intrinsic and extrinsic modes of apoptosis [84]. In the periphery-active HIV, replication leads to differentiation to plasmablasts [64] and immature transitional B cells with an abnormal expansion of exhausted B-cell subpopulation.

Immature-transitional B-cells (CD19^+^ CD10^+^ CD24^high^ CD38^high^) represent a critical link between immature B-cells in the bone marrow and mature naïve B-cells, which differentiate into switched memory B-cells, and in turn become overrepresented. This B-cell subpopulation is increased during active HIV replication and has been shown to be related to high levels of viremia, in parallel with a decrease of memory B-cell subset in vertically HIV-infected children [63].

Plasmablasts (CD10^−^ and CD21^low^) show an increased expression of the cell cycle Ki-67 immune proliferation marker and of the death receptor CD95^+^, which have been reported to be related to apoptosis [61, 85]. In addition, a large proportion of expanded B-cells in HIV-viremic individuals includes a subset of “tissue-like,” exhausted B-cells expressing the Fc-receptor-like 4 (FCRL4) and low levels of CD21^+^ [86, 87]. This subset presents increased expression of multiple inhibitory receptors, altered expression of homing receptors, and reduced proliferative potential [63]. Expansion of all these subsets occurs during active HIV replication and may contribute to the development of suboptimal immune responses.

#### 5.1.3. Loss of Resting Memory B Cell

The persistent high viral replication has been reported to directly affect the B-cell compartment [88, 89]. HIV-infected children show a significant loss of relative and absolute numbers of CD27^+^ memory B cells [90]. The loss of mature B cells (CD19^+^ CD27^+^) impairs long-term maintenance of protective antibodies titers [91–93] and has been shown to persist despite successful HAART [92, 94, 95]. However, the loss of antigen-specific memory B-cell responses begins in the early stages of HIV infection and becomes increasingly evident as the infection progresses to the chronic stage. Indeed, we have reported [92] that numbers of measles-specific memory B cells declined rapidly in vertically infected children who started HAART later than 1 year after birth. Thus, early initiation of HAART may preserve the normal development and long-term maintenance of the memory B cells generated in response to childhood immunizations.

#### 5.1.4. IL-21 Downregulation

The IL-21/IL-21R pathway has recently been identified to play a critical role in the development and maintenance of memory B-cell responses. IL-21 is a T-cell-derived pleiotropic cytokine whose receptor (IL-21R) is expressed by NK, T and B cells, and it seems to play a key role in the activation, expansion, and survival of these cells [96, 97]. Impaired antigen specific IL-21 secretion by CD4^+^ T cells in progressive HIV infection has been reported [98]. In a recent study, upregulation of IL-21R on B-cells and IL-21 secretion were proposed as a hallmark to identify responders to H1N1 vaccination among HIV-infected adults [98]. However, since IL-21 is produced mainly from CD4^+^ cells, in particular from T follicular helper cells [99], reconstitution of this subset of CD4 T cells with HAART in HIV-infected patients may be critically important to restoring B-cell function. Comparable studies addressing the role of IL-21 in determining the effectiveness of vaccine-induced immune responses in HIV chronically and acutely infected children will contribute to further understand the mechanisms leading to suboptimal immune response in this very particular group of HIV-infected patients. Furthermore, IL-21 production appears to be crucial for antiviral responses. For instance, it has been recently reported in a mouse model, that younger mice, which presented reduced IL-21 levels, showed a suboptimal generation of HBV-specific CD8^+^ T-cell and B-cell responses [100].

#### 5.1.5. Downregulation of BAFF and APRIL

Many studies have identified T-independent mechanisms of modulation of antibodies production [101–103]. Pallikkuth et al. have recently found that two innate immune factors, the B-cell Activating Factor (BAFF) and A Proliferation Inducing Ligand (APRIL), were present at lower levels in HIV-infected adults on HAART not responding to H1N1 vaccine compared to responders [104]. Future efforts are required to dissect the role of T independent immune factors including BAFF and APRIL, with the aim to limit Ab response failure to vaccinations particularly in clinical settings of impaired T-cell Help such as HIV/AIDS, in both adults and infants.

#### 5.1.6. Loss of Maintenance of Protective Antibody Titers

Both primary and secondary Ab responses are impaired during HIV infection, leading to loss in the maintenance of protective antibody titers which may not be restored by HAART [105]. A recent meta-analysis identified 38 studies in which immune-specific responses were analyzed in vertically infected children [106]. In general, fewer HIV-infected children with achieved protective immunity might experience greater and more rapid waning of protective immunity. Loss of protective humoral responses has been reported in infected children despite successful HAART [74, 77]. Persistence of measles antibody titers (MAt) (>50 mIU/mL cut-off value for specific immune response) [107], represents a good experimental model to analyze the longevity of humoral responses in HIV vertically infected children [108]. Protective levels of MAt have been shown to correlate with memory B-cells numbers in healthy individuals and to persist for the entire life span after successful immunization [107]. Generally, impaired development and maintenance of protective MAt has been reported in vertically infected children [92, 107]. As previously discussed, the decline of resting memory B-cells that occurs during the early stages of HIV infection may be an important pathogenic mechanism linked to the low level of measles-specific antibodies reported in HIV-infected children. In addition, persistent viral replication at the time of immunization can impair the generation and maintenance of protective Ab titers. Reduced or absent protective Ab titers versus HBV [109] and A H1N1 [110] have been reported in HIV-infected children, who were viremic at the time of vaccination.

 Loss of total CD4^+^ cells count can also play a crucial role in this process. Noteworthy, emerging data point out the importance of specific CD4^+^ T cell subsets depletion such as follicular CCR5 helper cell [99], Tregs, and Th17 which can play a crucial role in the induction and maintenance of protective immune responses [111]. Reduction of follicular CCR5 helper cell has been recently related to lower antibody responses to H1N1 vaccination in HIV-infected individuals. Similarly, studies on pandemic H1N1 have shown that specific cellular immune-mediated surveillance is crucially modulated by Th17 and Tregs. Cellular immune response to the influenza virus appears to be lower in HIV-seropositive patients than general population [111].

Reduction of Th17/Treg balance was found in untreated HIV-infected adults and increased after twelve months of HAART initiation as well as the IL-17 level [112]. Similarly, a significant loss of IL-17 producing PBMC was also found in viremic HIV-infected children [113]. A longitudinal assessment of Th17 cell dynamic in the peripheral blood is needed in order to determine the influence of timing of HAART initiation in preserving such subset in HIV-infected children.

### 5.2. Timing of Treatment

#### 5.2.1. Timing of HAART Initiation and Specific Responses to Vaccination

**Figure 1:**
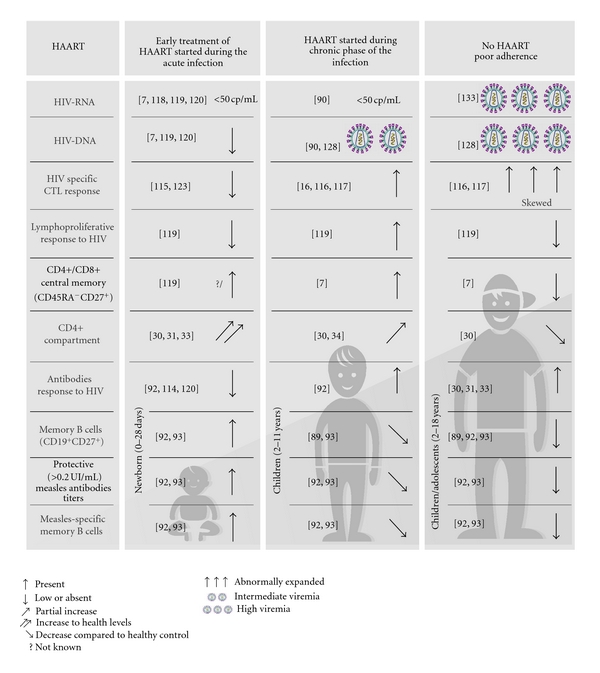
How timing of HAART initiation impact on B- and T-cell compartment and on viral replication.

The loss of specific B-cell memory clones occurs during the early stages of HIV infection. Thus, timing of HAART initiation seems to be crucial, since it may result in a different grade of disturbance of B-cell immune reconstitution, especially in pediatric patients. However, very few studies have addressed this issue in childhood [92, 114]. We previously showed that children who began HAART within the first year of life presented levels of memory B-cell percentages comparable to healthy uninfected age-matched controls. Conversely, children who began treatment after the first year of life had significantly lower percentages of memory B-cells compared to healthy controls [92]. Furthermore, it was shown that maintenance of resting memory B cell number is related to a better preservation of B-cell memory functionality. Indeed patients treated early maintained protective levels of measles and tetanus antibody titers, mirroring the preservation of B-cell memory repertoire as suggested by ELISpot analysis (Figure 1).

Similarly, early initiation of HAART in infected adults has been shown to prevent irreversible B-cell compartment damage resulting in a more functional profile of memory B-cell responses to HIV and non-HIV antigens when compared with chronic-treated HIV-infected individuals [108]. These data suggest that an early initiation of HAART is needed to preserve B cell compartment and obtaining an optimal response upon vaccination [115]. Whether or not HIV vertically infected children starting HAART later than the first year of life need different vaccine schedule is still debated [92, 105].

#### 5.2.2. Timing of HAART Initiation and Specific HIV Response

Timing of HAART initiation can influence the development of HIV-specific immune responses [116–120]. Some authors suggest that a rapid suppression of viral replication during a period of relative immunological immaturity might critically hamper the priming and expansion of virus-specific immune responses in vertically infected children. In fact, it has been shown that vertically infected children starting HAART within the first 3 months achieved long-term viral suppression, but did not develop HIV-specific antibodies and remained seronegative [30, 121–123]. In line with these data, initiation of HAART during acute HIV infection in adults results in incomplete or absent specific Ab HIV responses [124]. In addition, absent or suboptimal HIV-specific lymphoproliferative and cytotoxic immune responses have been reported by several authors [117, 118, 125–128]. Thus, the control of viremia achieved during different phases of the infection (acute versus chronic) can result in the development of suboptimal or strong HIV-specific immune responses (Figure 1). 

## 6. Conclusive Remarks

Vaccination is certainly among the most effective clinical interventions aimed at preventing infectious disease. However, the immune responses obtained following immunization are inadequate in HIV-infected children who present a suboptimal immune reconstitution. The capacity of the immune individuals to respond to vaccinations depends on diverse immunogenetic factors, but also on the degree of immunologic impairment at the time of immunization. The majority of vertically HIV-infected children who have access to antiviral treatment nowadays live into adolescence and adulthood. A large proportion of these children might be susceptible to vaccine-preventable childhood disease. Today, many uncertainties remain about the optimal strategies for identifying such susceptible individuals, and for offering them sustained protection through an appropriate immunization schedule, both in terms of timing and number of vaccine doses [129].

According to the findings presented below, we can highlight two main factors. First, HAART should be administered to children during the primary HIV infection to preserve the normal development of specific immune responses. Second, control of viremia should be achieved prior to performing any vaccination since HAART improves the capacity to establish and maintain long-term memory responses in individuals with HIV. 

Finally, we also believe that providing an in-depth understanding of the factors that regulate the development of protective immune responses is the sole pathway for rationally devising novel vaccination strategies against emerging infections, particularly in a large group of immune compromised patients, where maintenance of protective immunity clearly remains a major clinical challenge.

## Figures and Tables

**Table 1:** Factors leading to suboptimal immune reconstitution in vertically HIV infected children.